# The Evolution of Human Handedness

**DOI:** 10.1111/nyas.12047

**Published:** 2013-05-06

**Authors:** Jeroen B Smaers, James Steele, Charleen R Case, Katrin Amunts

**Affiliations:** 1 Department of Anthropology, University College LondonLondon, United Kingdom; 2 Department of Genetics, Evolution and Environment, University College LondonLondon, United Kingdom; 3 University College London, Institute of ArchaeologyLondon, United Kingdom; 4 Florida State University, Department of PsychologyTallahassee, Florida; 5 Research Center Jülich, Institute of Neuroscience and Medicine INM-1, and JARA-BrainJülich, Germany; 6 Departments of Psychiatry, Psychotherapy, and Psychosomatics, University Hospital AachenAachen, Germany

**Keywords:** laterality, primates, cerebellum, prefrontal cortex, tool use

## Abstract

There is extensive evidence for an early vertebrate origin of lateralized motor behavior and of related asymmetries in underlying brain systems. We investigate human lateralized motor functioning in a broad comparative context of evolutionary neural reorganization. We quantify evolutionary trends in the fronto-cerebellar system (involved in motor learning) across 46 million years of divergent primate evolution by comparing rates of evolution of prefrontal cortex, frontal motor cortex, and posterior cerebellar hemispheres along individual branches of the primate tree of life. We provide a detailed evolutionary model of the neuroanatomical changes leading to modern human lateralized motor functioning, demonstrating an increased role for the fronto-cerebellar system in the apes dating to their evolutionary divergence from the monkeys (∼30 million years ago (Mya)), and a subsequent shift toward an increased role for prefrontal cortex over frontal motor cortex in the fronto-cerebellar system in the *Homo*-*Pan* ancestral lineage (∼10 Mya) and in the human ancestral lineage (∼6 Mya). We discuss these results in the context of cortico-cerebellar functions and their likely role in the evolution of human tool use and speech.

## Introduction

Lateralization in human motor functioning is often considered as a principal factor explaining the exceptional capacity of humans to learn complex motor skills in a wide range of tasks. Lateralization in motor behavior and in its underlying neural systems is, however, not unique to humans, with extensive evidence demonstrating lateralization in primates,^1^–^3^ nonprimate mammals,^4^–^6^ birds,^7^,^8^ fish,^9^,^10^ reptiles,^11^,^12^ and amphibians^13^,^14^ (e.g., pawedness in toads,^15^ footedness in birds,^16^ and handedness in fish^17^). Considering the evidence for an early vertebrate origin for lateralized motor behavior and its close links to neural structural asymmetries,^18^,^19^ human lateralized motor functioning could be considered in a broad primate evolutionary context of neural organizational patterns with possibly deep evolutionary roots. Here, we aim to elucidate aspects of the neural evolutionary origin for complex motor learning and its lateralization, in the context of millions of years of divergent primate evolution.

We focus on quantifying the evolution of a brain system fundamental to motor control (the fronto-cerebellar system) across 46 million years of divergent evolution in anthropoids. The brain is organized as a distributed system, with different anatomically and functionally connected areas interacting in coordination to produce complex behaviors. The acquisition and adaptation of complex manual motor sequences involves activation of a frontoparietal praxis network involved in hand manipulation skills,^20^,^21^ as well as a frontocerebellar–basal ganglia network involved in novel motor sequence learning.^22^–^25^ Within the fronto-cerebellar network, the lateral hemispheres of the cerebellum receive input exclusively from the cerebral cortex projecting to the frontal motor and prefrontal areas via the dentate nucleus.^26^ The traditional (and empirically well-supported) theory of cerebellar function is that it encodes and continuously refines input–output relationships between motor commands and their consequences^27^ in both feed-forward and inverse feedback models of ongoing movements during action execution.^28^ In relation to its prefrontal projections, the cerebellar cortex simulates the way in which the outputs of prefrontal areas are processed, allowing it to issue feed-forward commands of correction signals back to the frontal lobe circuits.^29^ This neural system crucially underlies the process of motor learning and allows the development of motor plans that are not coded for limb-specific movements, but for the goal of an action.^30^ Functional neuroimaging studies of complex forms of motor learning confirm this interpretation of the role of the prefronto-cerebellar system in motor learning, by indicating that in the initial stages of learning, prefrontal processes control complex action execution, but that when the motor sequence is learned as a specialized automatic execution, cerebellar activation increases and prefrontal activation decreases.^31^–^33^ Considering the function of this prefronto-cerebellar system in the context of human evolution, we can hypothesize that its elaboration under natural selection could explain humans’ exceptional capacity to acquire, and continuously and dynamically adapt, complex forms of motor behavior.

Evidence for laterality in the fronto-cerebellar system primarily comes from human studies. The distributed cortical network involved in complex tool use is functionally left biased,^20^ and both prefrontal cortex^34^ and frontal motor areas^35^,^36^ have been demonstrated to be structurally lateralized in relation to language processing and handedness (e.g., neural asymmetry in primary motor cortex corresponds to behavioral lateralization in hand preference). Cerebellar directional asymmetry of size has been observed for lobules III and IV (left < right) and VI (left > right),^37^ possibly also related to handedness.^38^ Laterality is thus an additional feature of the prefronto-cerebellar system in humans, and may underlie humans’ exceptional capacities in tool use and language.

Despite comparative evidence suggesting increased prefrontal input to the cortico-cerebellar system^39^,^40^ and of its lateralization in human evolution, there is only limited information on the evolutionary history of these patterns of brain system connectivity. The main reasons for this are that previous studies have compared humans with a maximum of three nonhuman primate species (without consideration of their phylogenetic relatedness), and that methods to infer detailed evolutionary pathways for all branches in a phylogenetic tree have only recently become available.^41^,^42^ Reconstructing the detailed evolutionary history of the prefronto-cerebellar system and of its structural lateralization is of crucial importance: this will both provide more detailed information on the selective pressures that have defined its adaptive role in primate behavior, and enable assessment of the deeper evolutionary history of its structural lateralization.

Our previous work on this aspect of brain system evolution^43^ demonstrated a selective and correlated expansion of both frontal cortex and the cerebellar hemispheres at the dawn of the ape and great ape radiations. Here, we extend our previous work by differentiating between prefrontal (PF) and frontal motor areas (FM) within the frontal cortex and by delineating the part of the cerebellar hemispheres that has the closest functional association with prefrontal cortex, i.e., the posterior lobe of the cerebellar hemispheres (PCH).^44^–^46^ We further differentiate between the left and right hemispheres in each case, allowing inference of the evolution of laterality in the fronto-cerebellar system. We have collected information for 16 extant primate species, and quantified evolutionary rates of hemisphere-specific volumetric changes in the PF, FM, and PCH along individual branches of the primate phylogenetic tree. We aim to infer the evolutionary origin of a hypothesized shift from a predominantly frontal motor to a predominantly prefrontal involvement in the cortico-cerebellar system, and to examine the possible association of that shift with increased structural lateralization.

## Materials and methods

### Brain data

We examined both hemispheres of 29 individuals from 16 anthropoid species (see Table 1). Data consist of serially sectioned brains from the Stephan, Zilles, and Zilles–Amunts collections^47^ housed at the C. & O. Vogt Institute for Brain Research (University of Düsseldorf, Germany). Volumetric data for frontal motor areas and prefrontal cortex were taken from our previous work,^48^,^49^ where they were measured using a delineation protocol involving a bootstrap approach of estimating cumulative volumes at successive slice intervals along the anterio–posterior and posterior–anterior axes of the cytoarchitectonically defined frontal lobe. Data presented here indicate the cumulative volumes up to the 7th section interval of the anterior-posterior axis (PF) and posterior-anterior axis (FM) (Supporting Fig. 1).^50^

**Table 1:** Volumetric data (mL) used in the current analysis

			Left hemisphere			Right hemisphere		
Species	Individual	Brain size	PCH	FM	PF	PCH	FM	PF
*Homo sapiens* (human)	5,694	1,216.00	46.33	51.91	42.56	46.67	38.18	59.12
	6,895	1,110.00	51.04	26.02	57.46	50.98	31.81	51.76
	1,696	1,622.00	72.22	61.92	69.25	70.09	61.85	71.40
	14,686	1,437.00	47.55	58.08	59.01	46.19	49.08	77.04
*Pan troglodytes* (chimpanzee)	280	444.98	12.62	20.85	17.70	12.37	21.91	16.74
	497	378.00	15.17	14.44	11.52	15.17	13.69	12.57
*Gorilla gorilla* (western gorilla)	375	434.36	20.68	21.79	13.61	20.01	23.56	13.36
	8,214	376.00	10.40	13.27	8.07	11.19	14.14	7.83
*Hylobates lar* (gibbon)	1,203	98.36	3.61	3.78	3.02	3.70	3.61	3.08
	397	107.00	3.87	5.04	2.70	3.90	5.23	2.43
*Papio anubis* (olive baboon)	97	184.36	4.05	6.42	4.72	4.24	6.44	4.69
*Cercopithecus mitis* (blue monkey)	261	72.39	1.34	2.29	1.50	1.42	2.25	1.79
*Cercopithecus ascanius* (black-cheecked white-nosed monkey)	219	59.36	1.15	1.85	1.29	1.13	1.82	1.33
*Erythrocebus patas* (patas monkey)	1,341	93.73	1.55	3.22	1.88	1.60	3.48	1.82
	1,545	89.00	1.87	3.24	2.04	1.84	3.32	1.91
*Miopithecus talapoin* (talapoin monkey)	1,171	39.67	0.69	1.26	1.04	0.66	1.11	1.17
	1,201	38.32	0.57	0.98	0.79	0.57	0.97	0.73
*Nasalis larvatus* (proboscis monkey)	1,365	62.02	1.77	2.50	0.81	1.88	1.86	1.04
*Procolobus badius* (western red colobus)	213	75.97	2.04	2.61	1.81	2.11	2.69	1.84
*Alouatta seniculus* (red howler monkey)	1,184	45.17	1.00	1.53	0.88	1.08	1.58	1.60
*Ateles geoffroyi* (Central American spider monkey)	1,000	102.70	2.65	3.74	2.84	2.63	3.48	3.68
*Lagothrix lagotricha* (Humboldt's woolly monkey)	1,571	88.16	2.16	3.25	2.17	2.18	2.91	2.26
*Pithecia monachus* (monk saki)	1,180	32.82	0.69	0.96	0.45	0.71	1.02	0.95
*Cebus albifrons* (white-fronted capuchin)	1,200	77.03	1.72	2.47	1.74	1.65	2.75	2.01
	6,062	68.53	1.80	1.63	1.94	1.78	1.68	2.56

PCH, posterior cerebellar hemispheres; FM, frontal motor areas; PF, prefrontal cortex. Data for PCH were measured for the current analysis; data on FM and PF were taken from our previous work.^48^–^50^

### Comparative cerebellar anatomy

The cerebellum occupies only 10–15% of total brain volume in primates,^51^ but it contains roughly half of the brain's neurons.^52^ Although cellular organization is very uniform compared to the cerebral cortex,^27^ there is a clear differentiation between different parts of the cerebellum in terms of input–output relationships with other brain areas.^26^ The macro-anatomical subdivisions of the cerebellum across mammals involve 10 lobules^53^–^55^ (defined as I–X), which have been found to relate to distinct topographical cortical connectivity patterns.^45^,^46^ In particular, lobules V, VI, VIIb, and VIIIa have reciprocal connections with frontal motor areas, whereas portions of lobule VI and the entirety of crus 1 and crus 2 (subdivisions of lobule VII that make up an average of 40% of total cerebellar gray matter in humans^37^) have connections with the prefrontal cortex.^44^,^45^

### Delineation of the posterior cerebellar hemispheres

We delineated all lobules posterior to the primary fissure. This measurement comprises lobules VI–X, thus including the prefrontal projecting lobules VI–VII that encompass the majority of cerebellar gray matter volume posterior to the primary fissure (up to 64% in humans^37^). Our present delineation of the posterior cerebellar hemisphere differs from our previous delineation of the overall cerebellar hemispheres^43^ in that it focuses more specifically on prefrontal projecting lobules VI–VII. All measurements are presented in Table 1 (for example delineations, see Supporting Fig. 2). Volumes were computed using the Cavalieri procedure.^56^,^57^ Systematic samples from each brain were taken, the position of the first section was chosen randomly, and subsequent sections were chosen based on a regular sampling interval. Twenty or more sections per brain^58^ were used and digitized with a flatbed scanner at 800 dpi.

### Phylogenetic scaling

In any comparative analysis, raw data will consist of phylogenetically nonindependent data points (e.g., sampled by species); comparisons of such points need to be weighted for phylogenetic distance. Allometric analyses of comparative datasets therefore incorporate phylogenetic trees, to account for differences that are due to phylogenetic relatedness. We use phylogenetically reduced major-axis regressions with a likelihood-fitted lambda model to obtain residuals from regressions of brain structure size on the rest of brain size.^59^ “Rest of brain” was here defined as total brain size minus size of PCH, FM, and PF. These residuals are used as measures of the relative size of a particular brain structure. 60 Relative sizes of brain structures were then scaled using phylogenetically generalized least squares regressions.^61^ All analyses were performed in the *R* software environment. 62–64

### Evolutionary rates and inferring evolutionary history

We use an adaptive peak model of evolution to infer rates of change for individual branches along the tree of life.^41^–^43^ This model allows for rates of change to be different for each branch in a phylogenetic tree in response to the wanderings of adaptive peaks through phenotype space. We use the adaptive peak model as formalized in the method of independent evolution^41^,^42^ because it allows for the incorporation of more specific models such as Brownian Motion and Ornstein Uhlenbeck as special cases by collapsing its algorithms accordingly under relevant conditions. This formalization has further been shown to accurately estimate fossil values of brain and body size in primates, bats and carnivorans,^41^,^42^ supporting its validity in estimating evolutionary trends for brain structure sizes in primates.

Reconstructing rates of the evolution of traits allow identification of branches in the evolutionary tree associated with episodes of selective and correlated trait coevolution, which can be compared with the general scaling patterns revealed by phylogenetic regressions.^41^ Evolutionary trends on individual branches may align with or diverge from more general evolutionary patterns as different species follow different adaptive directions. Importantly, recognition of general scaling regularities simply identifies average trends across all species in a sample, and does not mean that changes in each branch of the phylogenetic tree necessarily exemplify them. The extent to which changes in particular branches align with the clade-general correlation pattern can be revealed by a comparison of evolutionary rates.^41^ This approach complements the use of phylogenetic regressions by allowing a more detailed investigation of the evolutionary history of trait coevolution along particular evolutionary branches.

## Results

### Comparative correlations between PCH, FM, and PF

**Figure 1:**
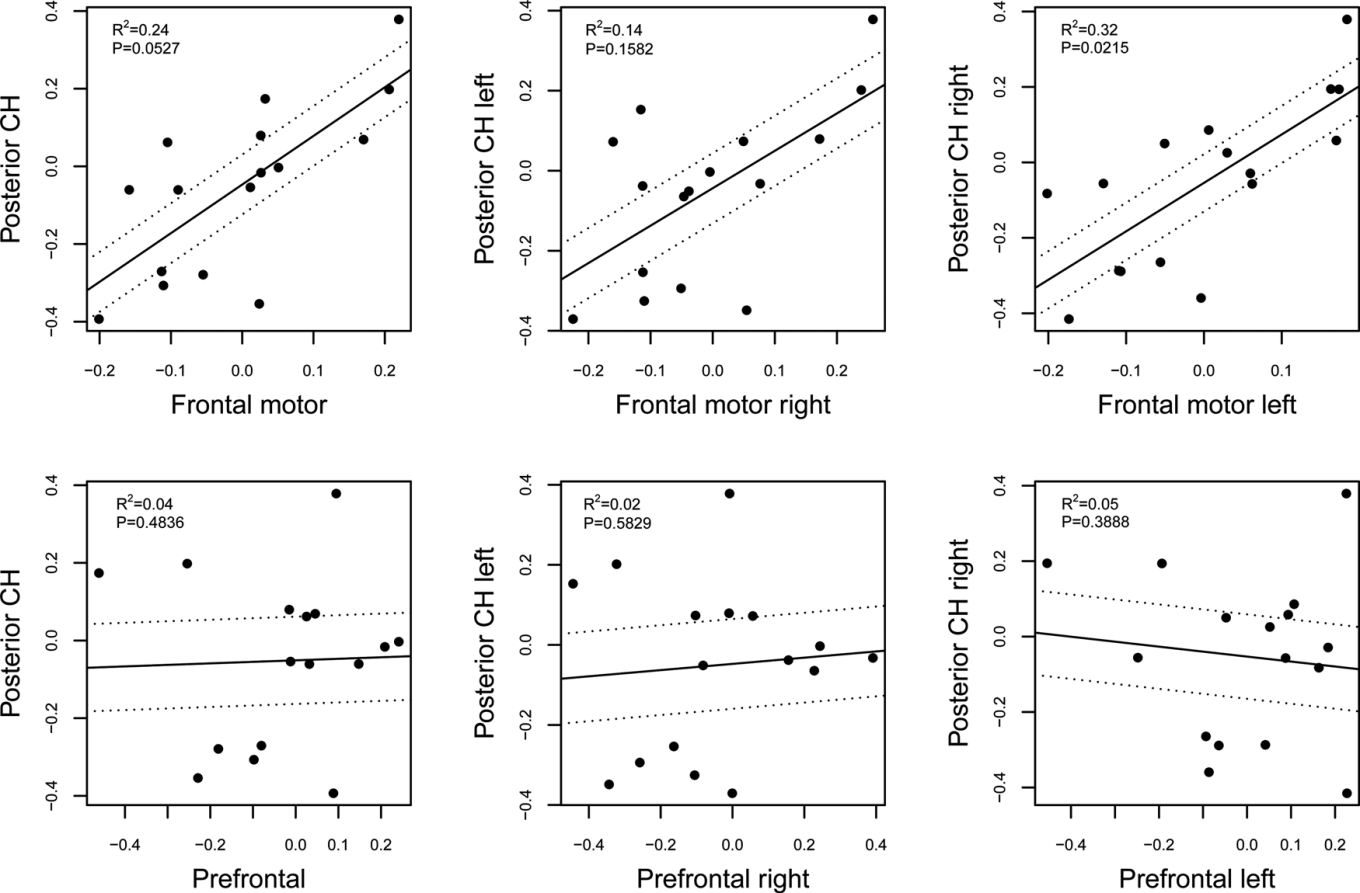
Results from a phylogenetically generalized least squares analysis of correlations in the relative size of the posterior cerebellar hemispheres, frontal motor areas, and prefrontal cortex. Hemisphere-specific regressions between frontal and cerebellar structures were performed contralaterally, because evidence suggests the importance of contralateral connections in the fronto-cerebellar system.^44^

Phylogenetically generalized least squares analysis with a maximum likelihood-fitted lambda model (Fig. 1) reveals a significant correlation between the relative sizes of PCH and of FM (slope 95% C.I. = 0.04:1.02, *R*^2^ = 0.24, *P* = 0.0527, λ = 1), but not of PCH and of PF (slope 95% C.I. = −0.57:0.27, *R*^2^ = 0.04, *P* = 0.4836, λ = 1). Considering evidence for contralateral cortico-cerebellar connectivity^44^ we further analyzed contralateral hemispheric correlations. Results demonstrate a significant correlation between right PCH and left FM (slope 95% C.I. = 0.17:1.23, *R*^2^ = 0.32, *P* = 0.0215, λ = 1), but not left PCH and right FM (slope 95% C.I. = −0.10:0.74, *R*^2^ = 0.14, *P* = 0.1582, λ = 1). No significant correlation was found between either right PCH and left PF (slope 95% C.I. = −0.56:0.21, *R*^2^ = 0.05, *P* = 0.3888, λ = 1) or left PCH and right PF (slope 95% C.I. = −0.46:0.26, *R*^2^ = 0.02, *P* = 0.5829, λ = 1).

### Laterality in PCH, FM, and PF

We investigated structural lateralization in PCH, FM, and PF by scaling hemisphere-specific volumes of each of these structures to rest of brain size (defined as brain size minus PCH, FM, and PF). Lateralization was assessed by comparing scaling coefficients (intercepts and slopes of the regression) for the left and right hemispheres. In this approach, significant results refers to comparisons of the 95% confidence intervals of the scaling coefficients from different analyses; if the scaling coefficient of one analysis lies outside the confidence interval of another analysis, it is considered to be significantly different at *P* < 0.05. Results are summarized in Table 2. For PCH and FM, there is no asymmetry in general scaling trends in primates. However, and consistent with previous analyses,^48^ for PF there is significant hyperscaling of the left compared to the right hemisphere, together with a significantly lower intercept.

**Table 2:** Results from a phylogenetically generalized least squares analysis of scaling of hemisphere-specific brain structures/areas to rest of brain size. Rest of brain size is here defined as total brain size minus the size of PCH, FM, and PF

	Left hemisphere			Right hemisphere		
	Slope	95% C.I.		Slope	95% C.I.	
PCH	1.64	1.46	1.82	1.63	1.44	1.82
FM	1.54	1.43	1.64	1.54	1.43	1.66
PF	1.82	1.64	2.00	1.63	1.45	1.82

	Intercept	95% C.I.		Intercept	95% C.I.	
PCH	−1.92	−2.35	−1.48	−1.89	−2.35	−1.43
FM	−1.64	−1.89	−1.38	−1.65	−1.93	−1.37
PF	−2.38	−2.82	−1.94	−1.90	−2.35	−1.44

### The evolutionary history of the fronto-cerebellar system

**Figure 2:**
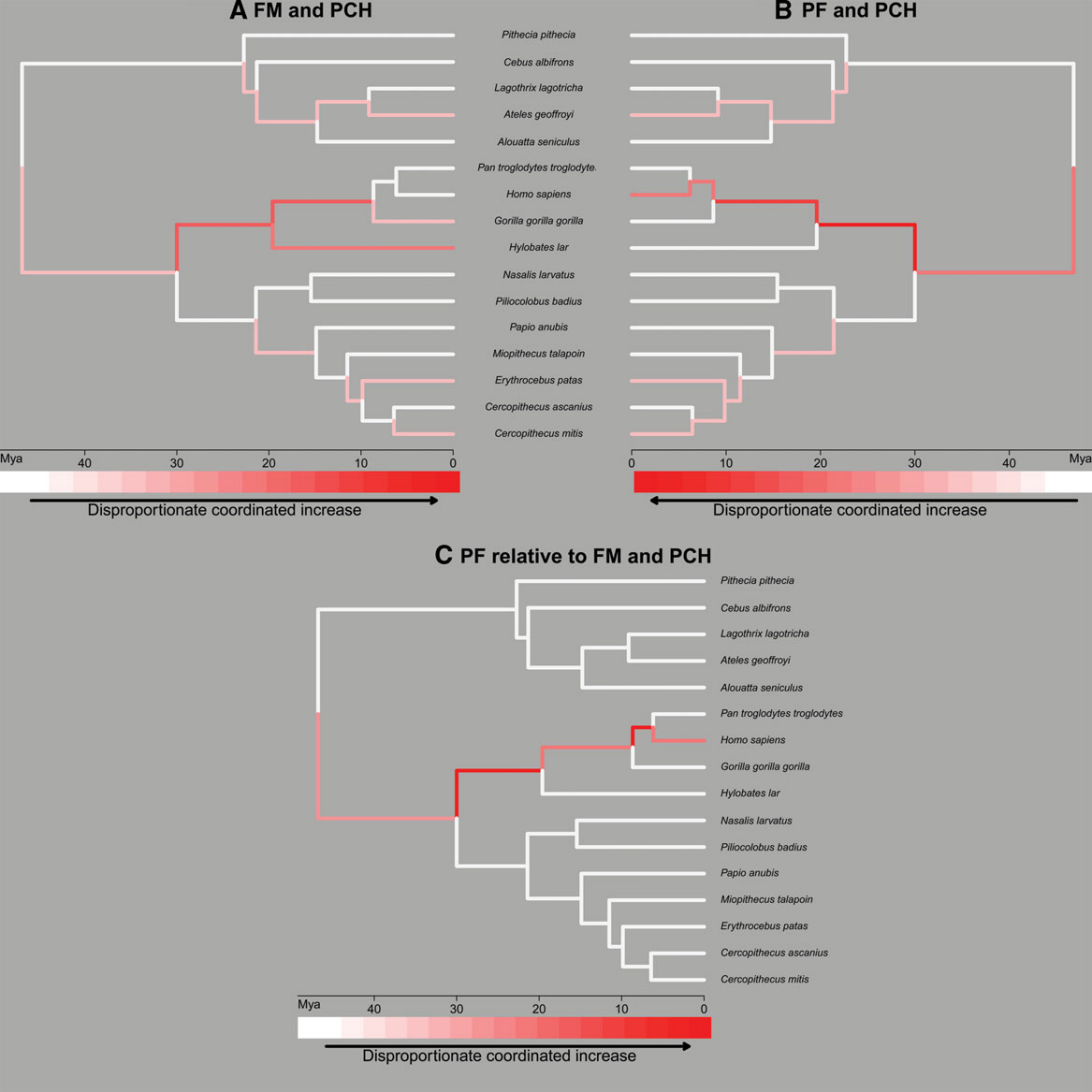
Comparative analysis of evolutionary rates of the posterior cerebellar hemispheres (PCH), frontal motor areas (FM), and prefrontal cortex (PF). Rates were compared for individual branches of the primate phylogenetic tree, allowing detailed inferences of the evolutionary history of fronto-cerebellar systems across 46 million years of divergent primate evolution.

We compared rates of evolution of PCH, FM, and PF relative to rates of evolution of rest of brain size, to investigate which branches align with or diverge from clade-general coevolutionary trends. We focus here on branches where our methods have reconstructed a disproportionate increase in PCH and either FM or PF, since this identifies branches in which the fronto-cerebellar system plays an unusually strong adaptive role. Disproportionate FM–PCH increase characterizes the ape (∼30 Mya) and great ape (∼20 Mya) ancestral branches, but the trend does not continue in branches leading specifically to *Pan* and to *Homo* (Fig. 2A). Disproportionate PF–PCH increase also characterizes the ape and great ape ancestral branches, but that trend continues in the *Homo*-*Pan* (∼10 Mya) ancestral branch and in the human (∼6 Mya) ancestral lineage (Fig. 2B).

To infer the evolutionary origin of a hypothesized shift from a predominantly frontal motor to a predominantly prefrontal involvement in the cortico-cerebellar system, we quantified the increase of PF relative to FM in relation to PCH. These results reveal directional selection for an increased PF contribution to the fronto-cerebellar system in branches leading from the Old World anthropoid ancestral lineage through to the human ancestral branch, with the most pronounced trends in the ape ancestral lineage and in the *Homo*-*Pan* ancestral lineage (Fig. 2C). Results further indicate that these trends are similar when assessing each contralateral cortico-cerebellar pattern (left PF/FM and right PCH versus right PF/FM and left PCH; Fig. 2 lists results across both hemispheres, and Supporting Figs. 3 and 4 give results for each contralateral pattern).

## Discussion

The fronto-cerebellar brain system plays a crucial part in the automatization of learned motor sequences and the incremental acquisition of movements into well-executed behavior.^25^,^27^,^65^–^67^ Reconstructing the evolution of this brain system will advance understanding of the evolution of humans’ exceptional motor capacities. To investigate the evolutionary history of humans’ increased (lateralized) prefrontal input to this brain system, we delineated relevant brain structures for 29 individuals from 16 different primate species, and quantified evolutionary rates on separate branches of the primate phylogenetic tree.

Our results indicate a significant contrast in the scaling of left versus right PF, but not of left versus right FM and PCH (Table 2). The lack of general trends for volumetric asymmetry in PCH across these 16 anthropoid species is consistent with findings of within-species variation in chimpanzees demonstrating “no population-bias in the lateralization of the cerebellum.”^68^ When considering hemisphere-specific correlations within the fronto-cerebellar system, we find stronger evidence for a left FM–right PCH coupling than for the contralateral pattern (Fig. 1). This is likely to be principally related to a frontal motor praxis system involved in primate hand manipulation skills. PF–PCH coevolutionary coupling at the level of correlations between contralateral hemispheres is not found consistently across the whole primate sample (Fig. 1).

To investigate lineage-specific patterns of brain reorganization,^41^ we quantified rates of evolution for each branch in the phylogeny. Although our sample included multiple individuals for several key species (including all the ape species represented) and are consistent with findings of the distribution of structural asymmetries in larger samples of specific species by other workers,^68^ future work should look to expand sample sizes for each species to increase the robustness of lineage-specific inferences. In Figure 2 we highlight branches for which we have reconstructed a coordinated and disproportionate increase in the size of the fronto-cerebellar system. This phylogenetic mapping reveals strong selective investment in both FM–PCH and PF–PCH at the dawn of the ape (∼30 Mya) and great ape (∼20 Mya) radiations, differentiating them from monkeys (Fig. 2A and 2B). A subsequent expansion of PF–PCH, but not FM–PCH, is indicated on the *Homo*-*Pan* ancestral branch (∼10 Mya) and in the human lineage (∼6 Mya). When comparing evolutionary rates for PF and FM, the ape ancestral branch, the *Homo*-*Pan* clade and the human ancestral lineage all appear to be characterized by shifts toward an increased role for PF in the fronto-cerebellar system (Fig. 2C).

These results, and the finding of left PF hyperscaling as a general trend in our sample, are consistent with observed structural brain asymmetries in humans and chimpanzees, absent in other nonhuman primates, for several relevant frontal and cerebellar areas,^3^,^68^–^73^ suggesting that at least part of the neural foundation for human complex motor behavior was present before the ancestral split with the lineage leading to chimpanzees. With increased selection for context- and goal-dependent, dynamic adjustment of learned motor plans (e.g., tool use), the prefrontal input to the cortico-cerebellar system may have become more pronounced and led to selection for increased lateralization. This suggestion is also supported by studies in chimpanzees demonstrating that individual variability in structural asymmetry of the PCH is related to the propensity to perform complex activities such as tool use and aimed throwing, and handedness for a tool-use task (termite fishing).^68^

Our finding that humans and chimpanzees share a preadaptation for increased prefrontal involvement in the fronto-cerebellar system, which continued in the human lineage but stabilized in the chimpanzee lineage, may shed light on the evolutionary role of the fronto-cerebellar system in tool use and vocal articulatory control, and on the differences in tool use and vocalizing abilities between these two species.

Humans and chimpanzees share the capacity to perceive the affordances of objects as potential tools^74^,^75^ and the ability to modify the kinetic energy produced in relation to the affordances of the task constraints.^76^,^77^ In both species, however, these capacities involve an experience-based learning process where increased experience results in increased efficiency. In other words, the ability to move from initial action execution of complex motor sequences to specialized automatic execution through experience plays a crucial role in nut-cracking in both humans and chimpanzees.^78^

Stone flaking, however, a bimanually coordinated task that became a habitual behavior within the hominin radiation, may require greater lateralization of hand function because it involves the two hands working at two different levels of resolution in a coordinated fashion to yield a common functional outcome (the hammering hand needs to be controlled in such a way as to transmit the appropriate amount of kinetic energy at impact with considerable accuracy at the point of percussion, whereas the postural hand has to rotate and adjust the position of a core to prepare for the following hammer strike, and stabilize the core against the shock of the blow^78^). Stout *et al*.^79^ have found increased frontal activation in stone flaking tasks, with site, lateralization and level of activation varying as a function of task complexity and task familiarity, but the extent of any similarities and differences with activation patterns in a nut-cracking task have not yet been studied in humans or in chimpanzees.

Humans are additionally distinguished from chimpanzees in possessing the capacity for articulate speech. Posterior cerebellar activation in language tasks has been found to be right lateralized and focused in lobule VI and crus 1,^80^ which, as noted above, are prefrontal-projecting areas. Fluent speech requires the serial ordering of phonemes and syllables, and it has been shown that preparation and production of more complex syllables and syllable sequences recruit left hemisphere inferior frontal sulcus, posterior parietal cortex, and bilateral regions at the junction of the anterior insula and frontal operculum, to supplement the more basic cortical and subcortical components of the speech production system.^81^ Activation patterns in a verbal motoric rehearsal task suggest the existence of a frontal (BA44/46) and superior cerebellar (lobuleVI/crus 1) articulatory control system,^82^ and there is increased PF activation with increased working memory loads in a speech motor control task.^83^ Thus, it is plausible that the expansion of the prefrontal system and of prefrontal-projecting cerebellar lobules in humans^39^ also relates to adaptations for articulate speech.

## Supporting Information

Disclaimer: Supplementary materials have been peer-reviewed but not copyedited.

**Figure S1.** Delineation examples for the posterior cerebellar hemispheres for *Homo sapiens*, *Hylobates lar*, *Cercopithecus mitis*, and *Cebus albifrons*.

**Figure S2.** Delineation protocol for PF and FM (from Refs. 48, 49, and 50). For each individual, the border between areas 3 and 4 is delineated using cytoarchtitectonic criteria. Cumulative volumes are computed along the anterio–posterior and posterio–anterior axes of the brain, anterior to the border of areas 3 and 4. (a) and (b) indicate the 4th and 17th sections of a human brain, (c) presents the dorsal view of the brain exemplifying the cumulative volumetric approach along the anterio-posterior axis.

**Figure S3.** As in Figure 2, results comprise the analyses of left cortical (PF and FM) and right cerebellar (PCH) hemispheres, and of right cortical (PF and FM) and left cerebellar (PCH) hemispheres.
