# Supplementary figures and images for: The Evolution of Human Handedness
Source: Ann N Y Acad Sci. 2013 May 6;1288(1):59–69. doi: 10.1111/nyas.12047 (PMC4298027; doi:10.1111/nyas.12047)

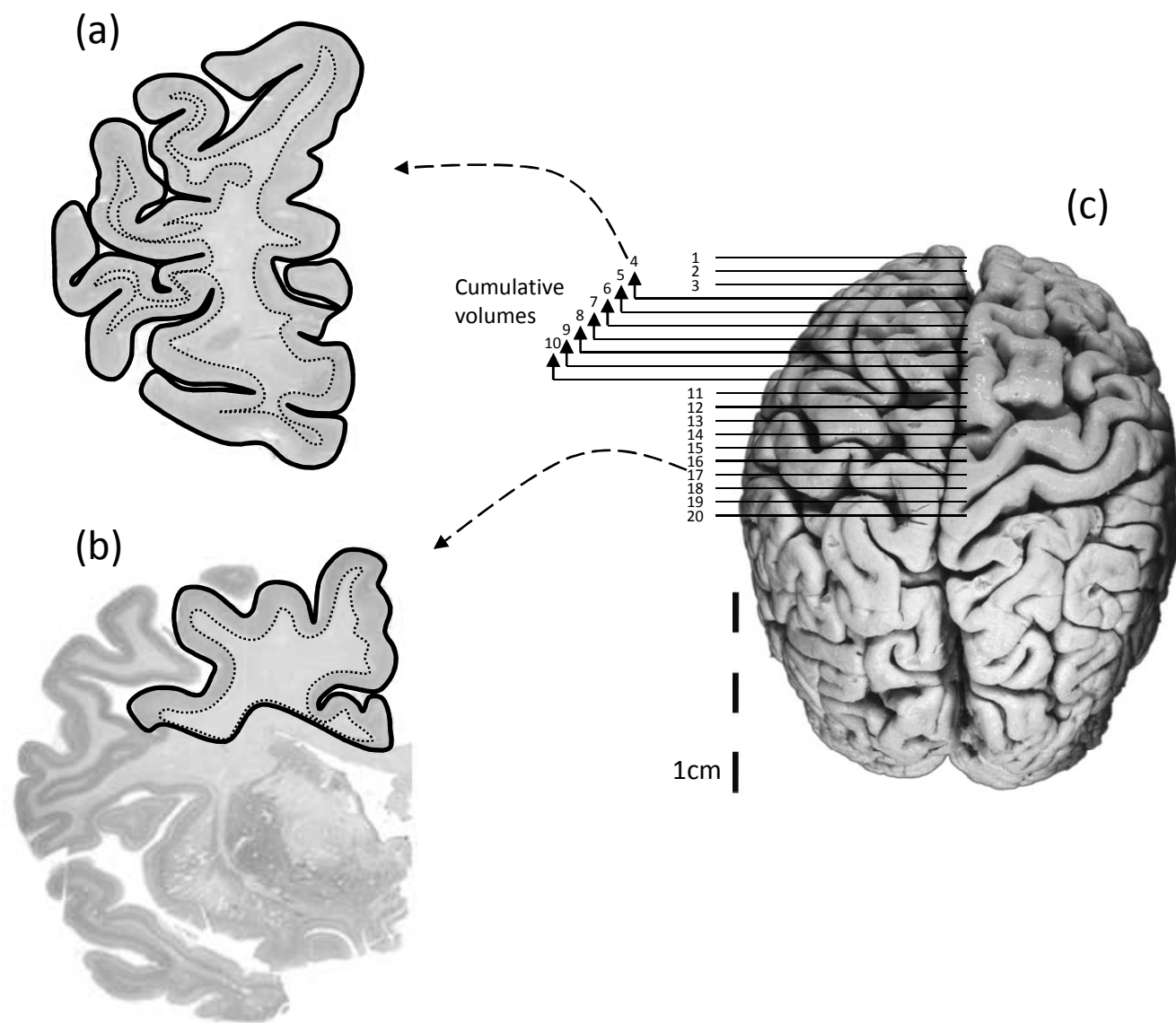

Supplement: Supplementary file 1 — Figure S1. Delineation examples for the posterior cerebellar hemispheres for Homo sapiens, Hylobates lar, Cercopithecus mitis, and Cebus albifrons. [file nyas1288-0059-sd1.pdf]

# *Homo sapiens*

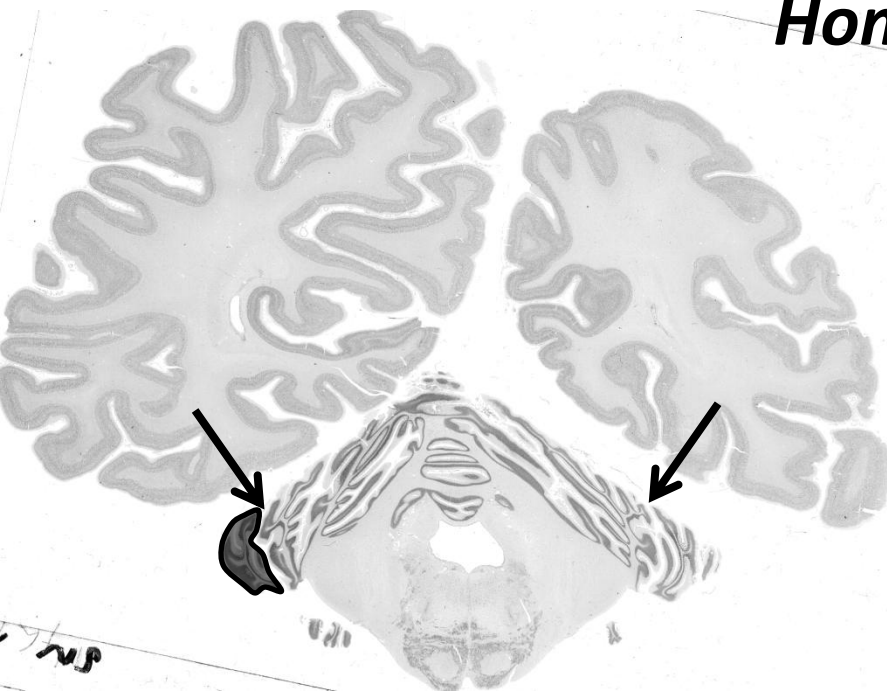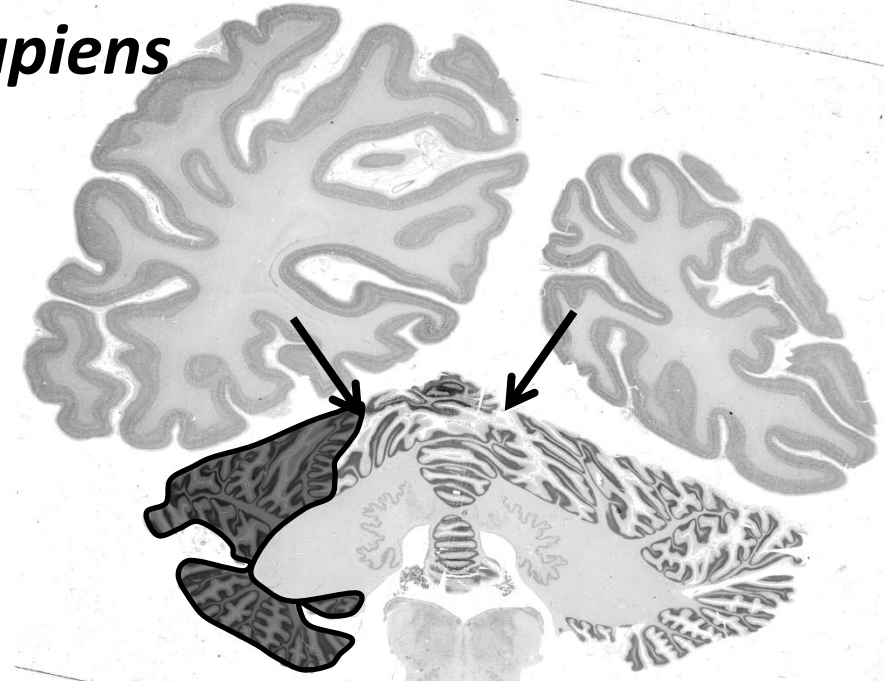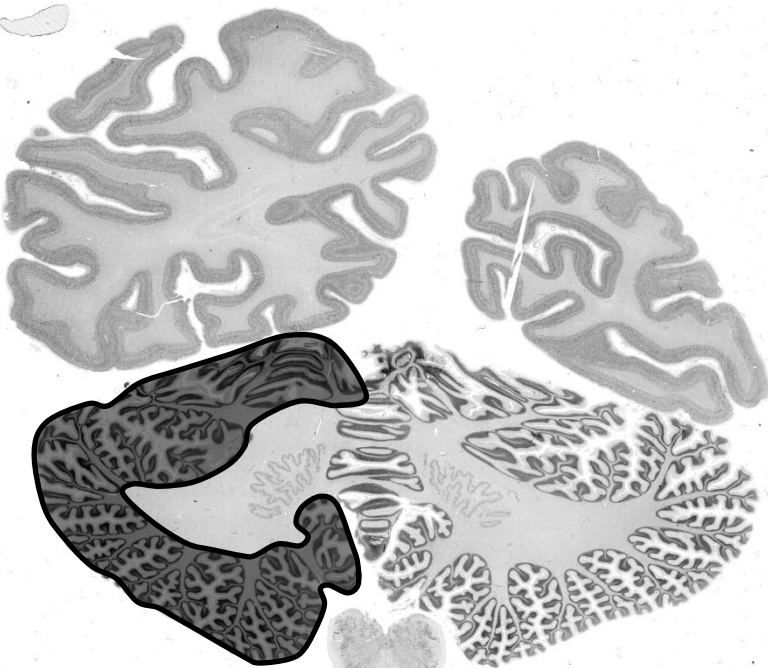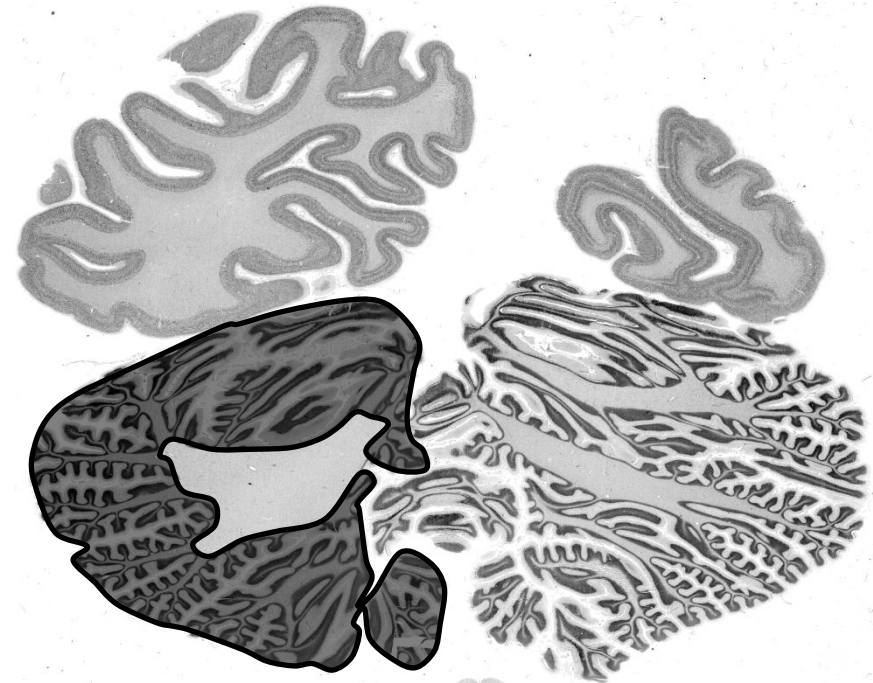

# *Hylobates lar*

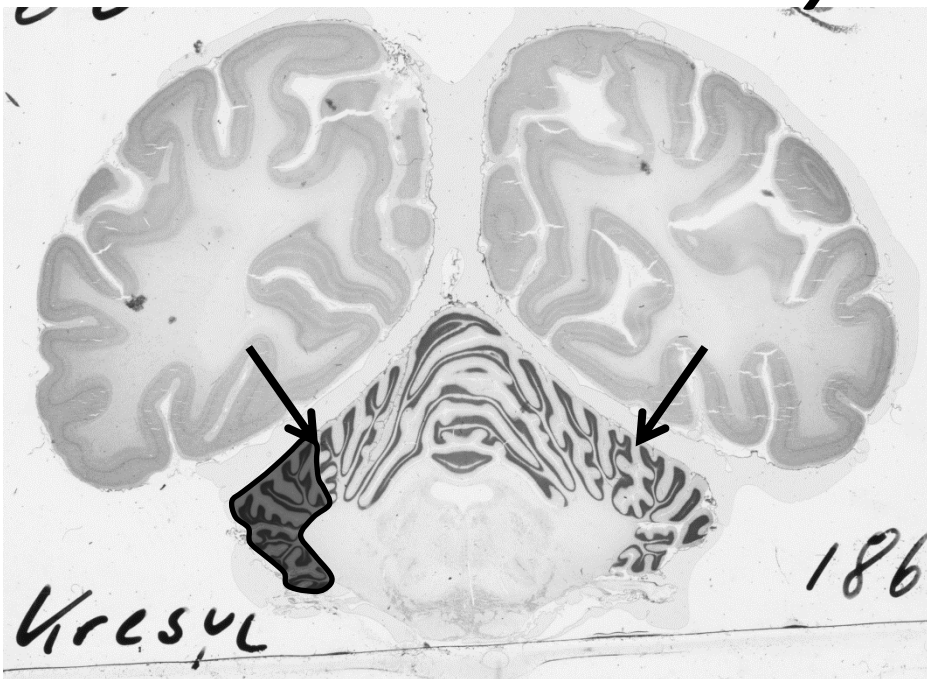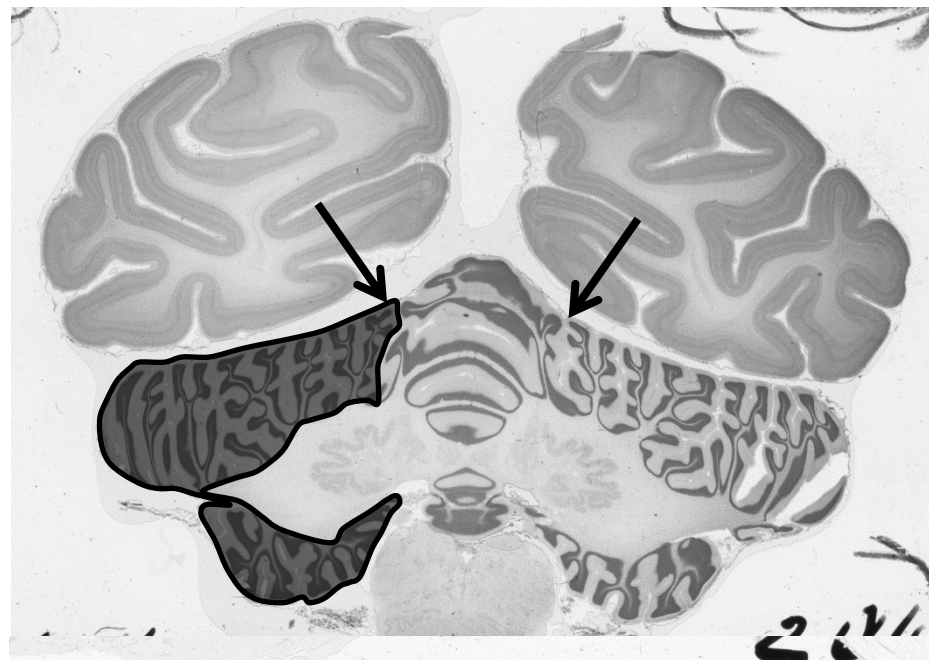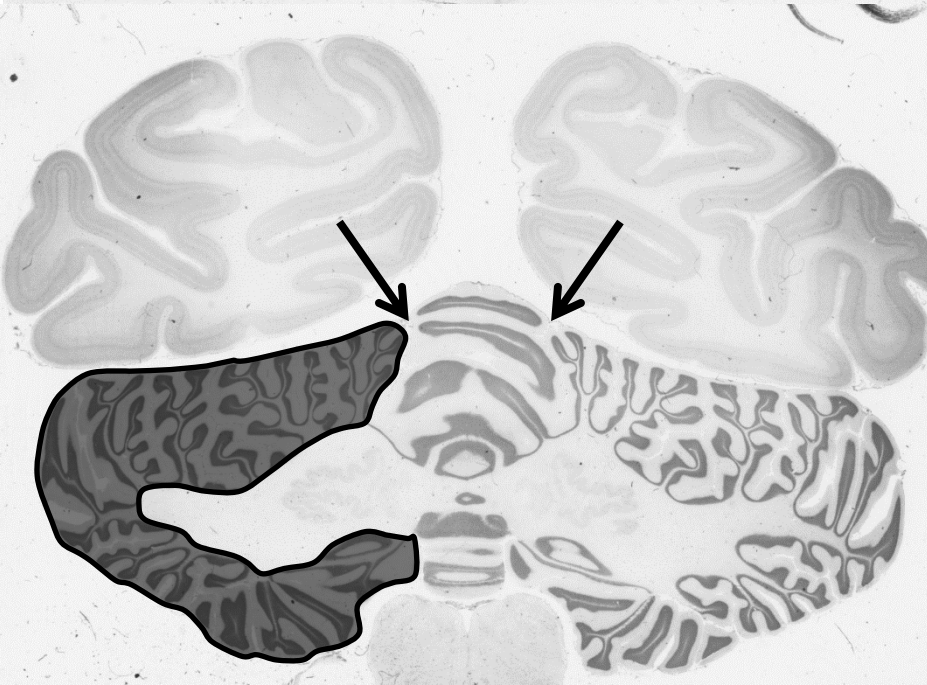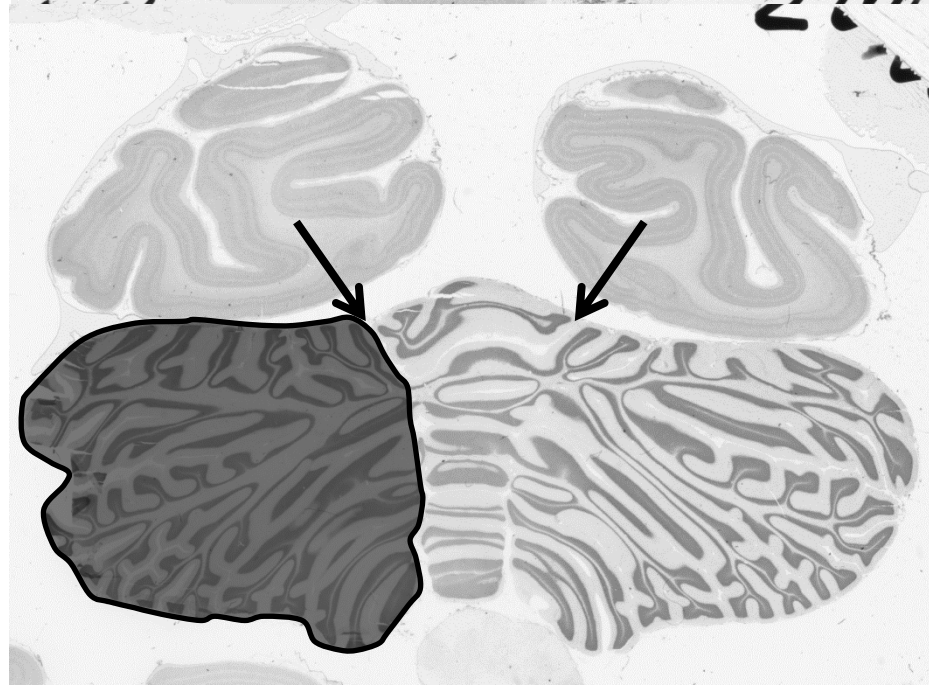

# *Cercopithecus mitis*

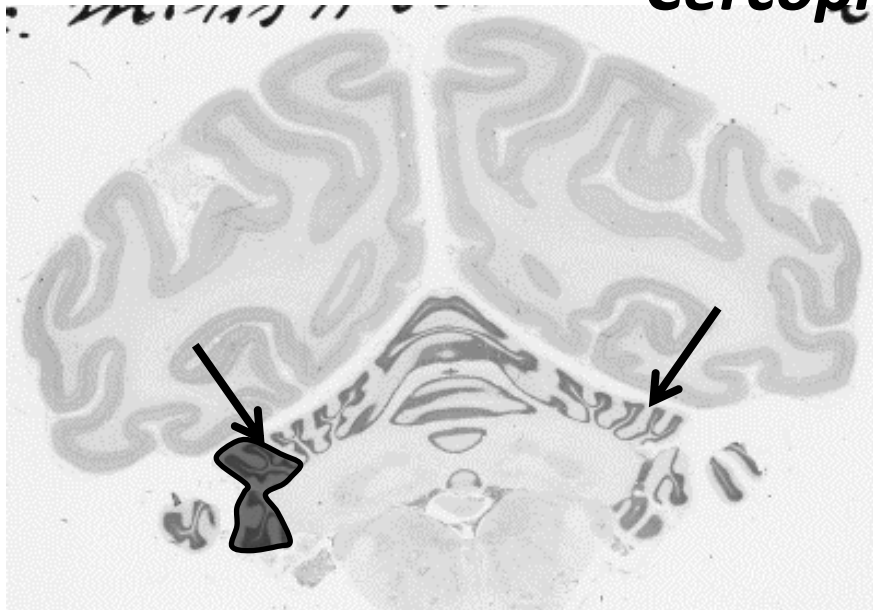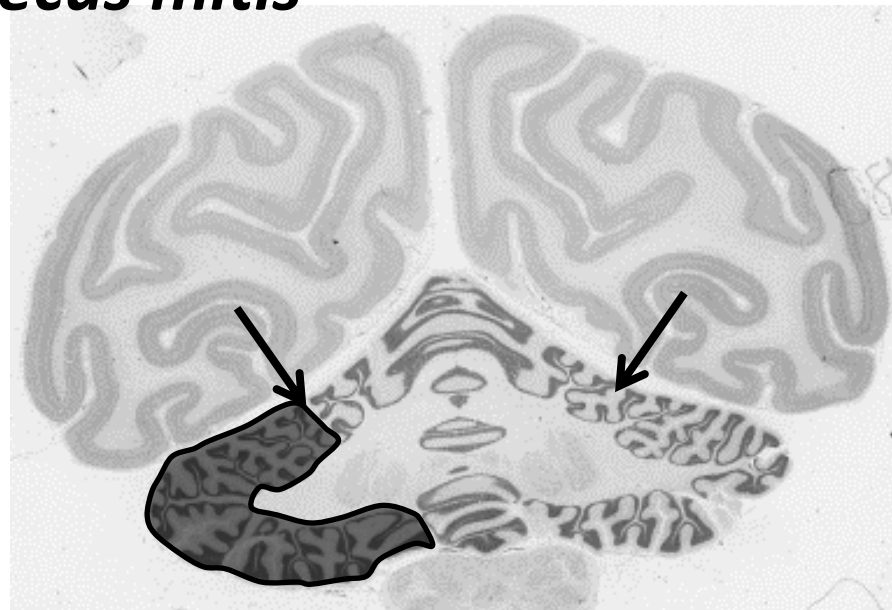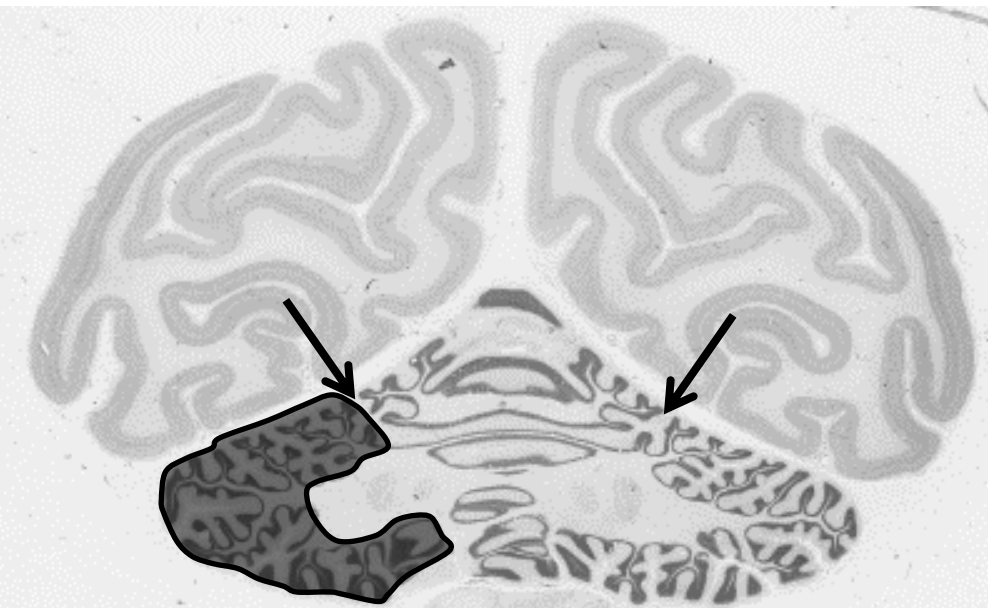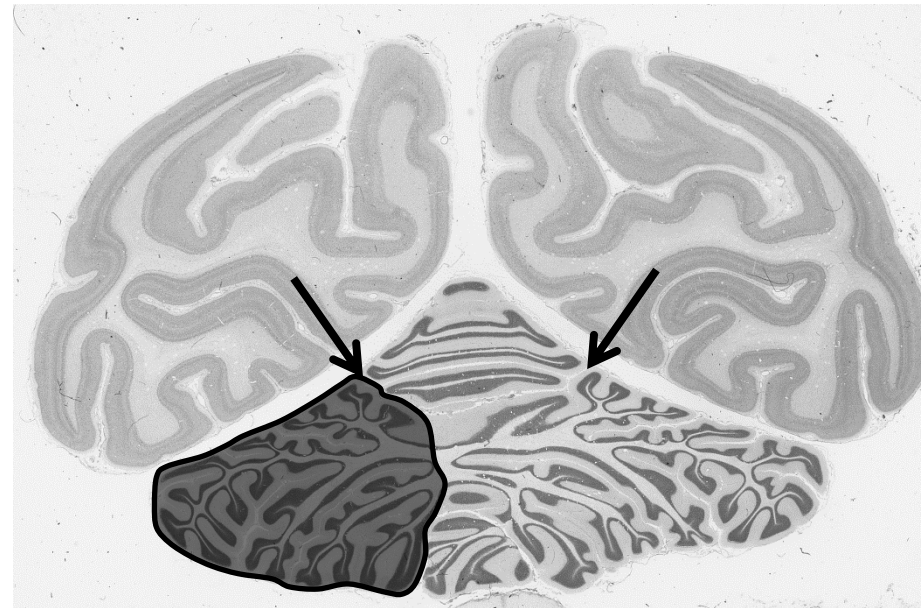

*Cebus albifrons*

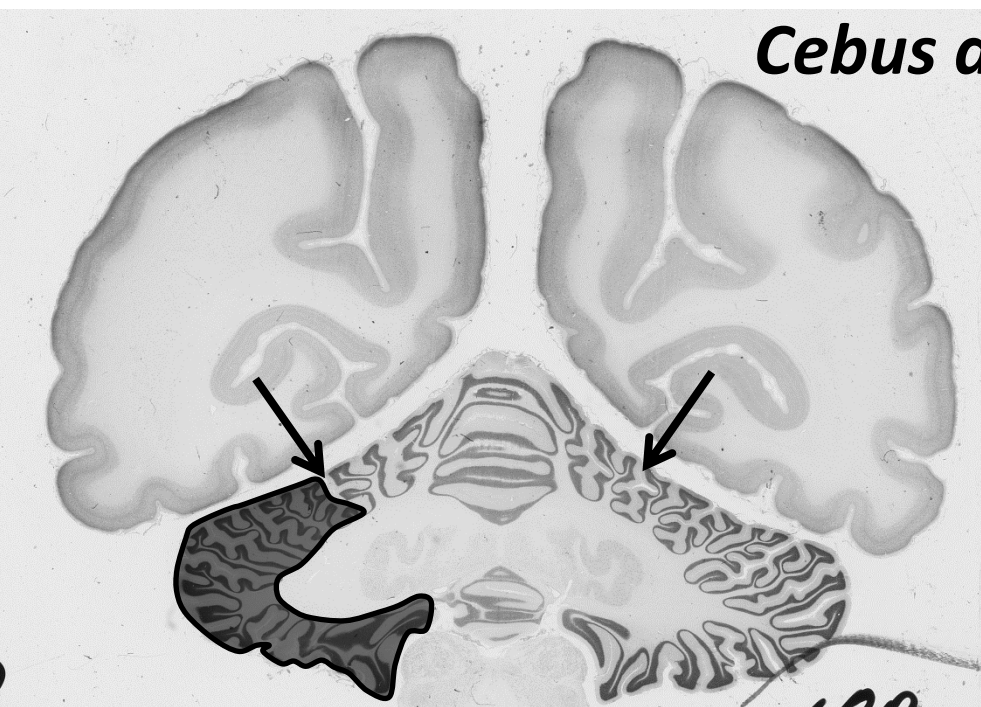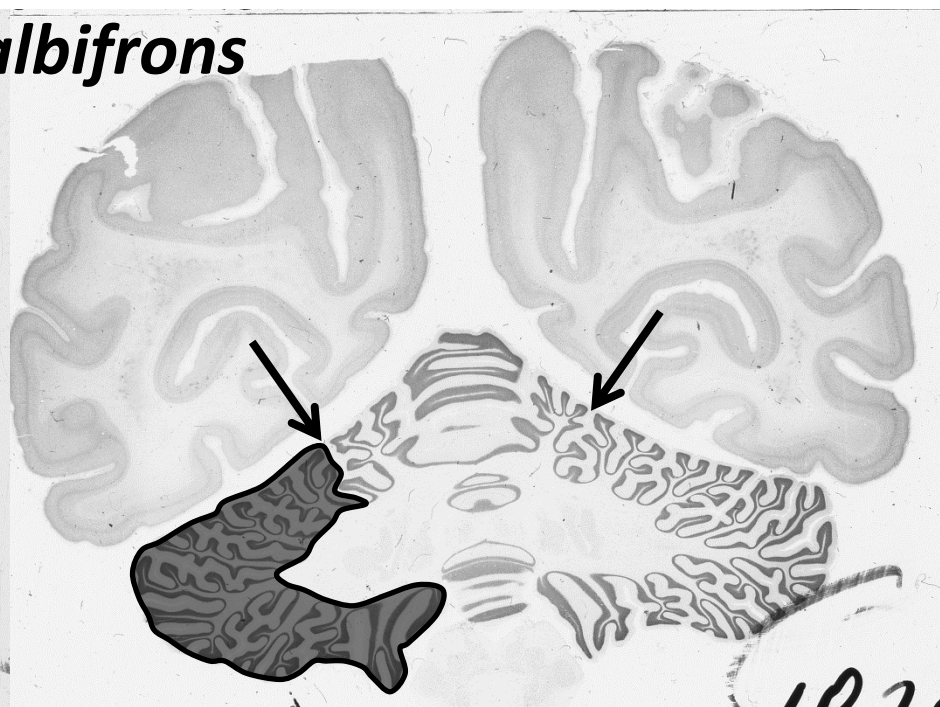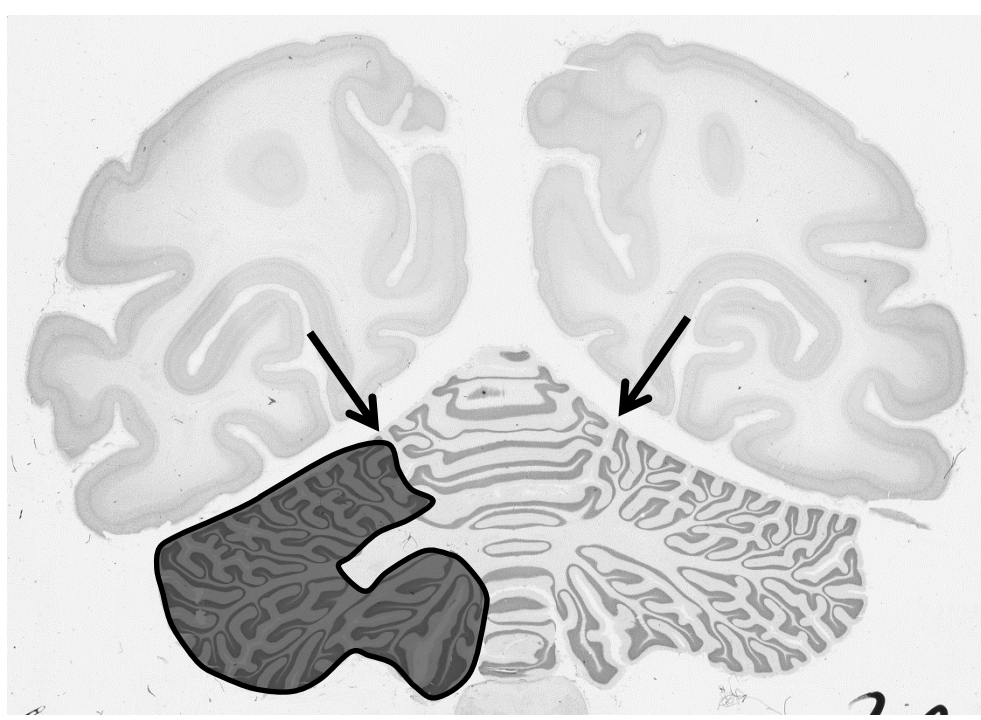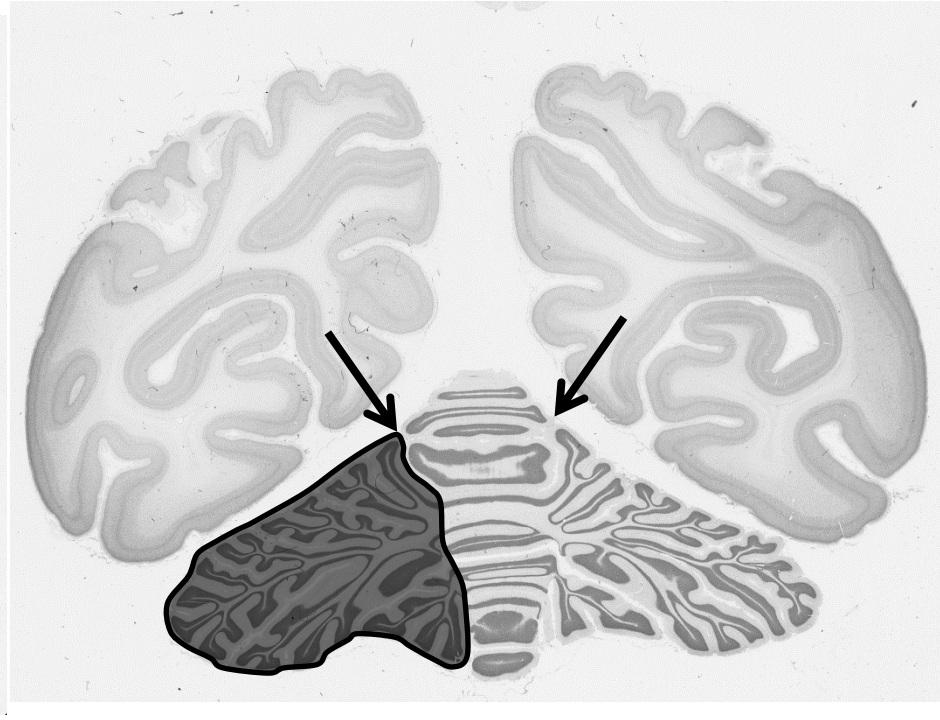

Supplement: Supplementary file 2 — Figure S2. Delineation protocol for PF and FM (from Refs. 48, 49, and 50). For each individual, the border between areas 3 and 4 is delineated using cytoarchtitectonic criteria. Cumulative volumes are computed along the anterio–posterior and posterio–anterior axes of the brain, anterior to the border of areas 3 and 4. (a) and (b) indicate the 4th and 17th sections of a human brain, (c) presents the dorsal view of the brain exemplifying the cumulative volumetric approach along the anterio-posterior axis. [file nyas1288-0059-sd2.pdf]

**(a) FM and PCH**

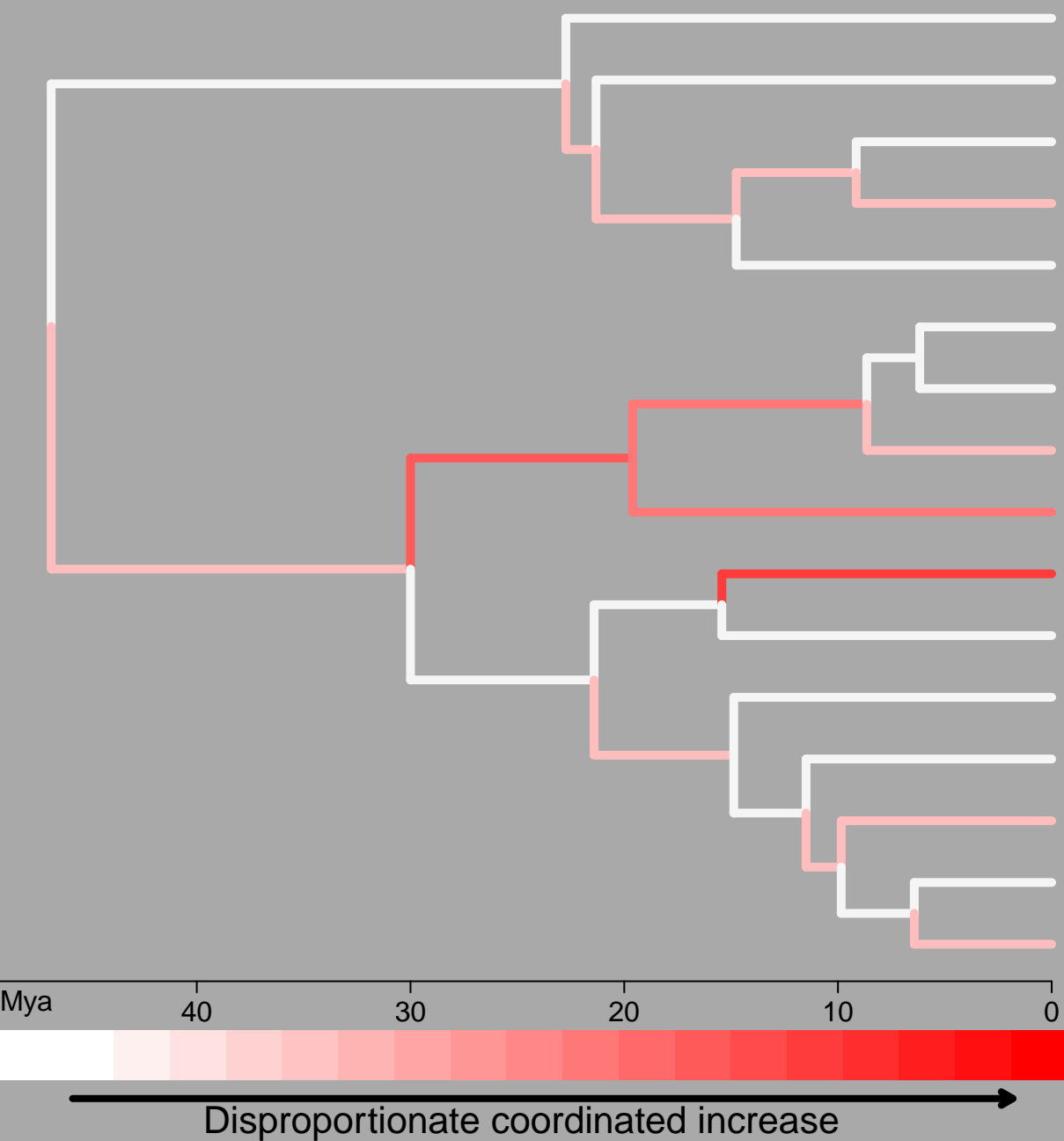

**(b) PF and PCH**

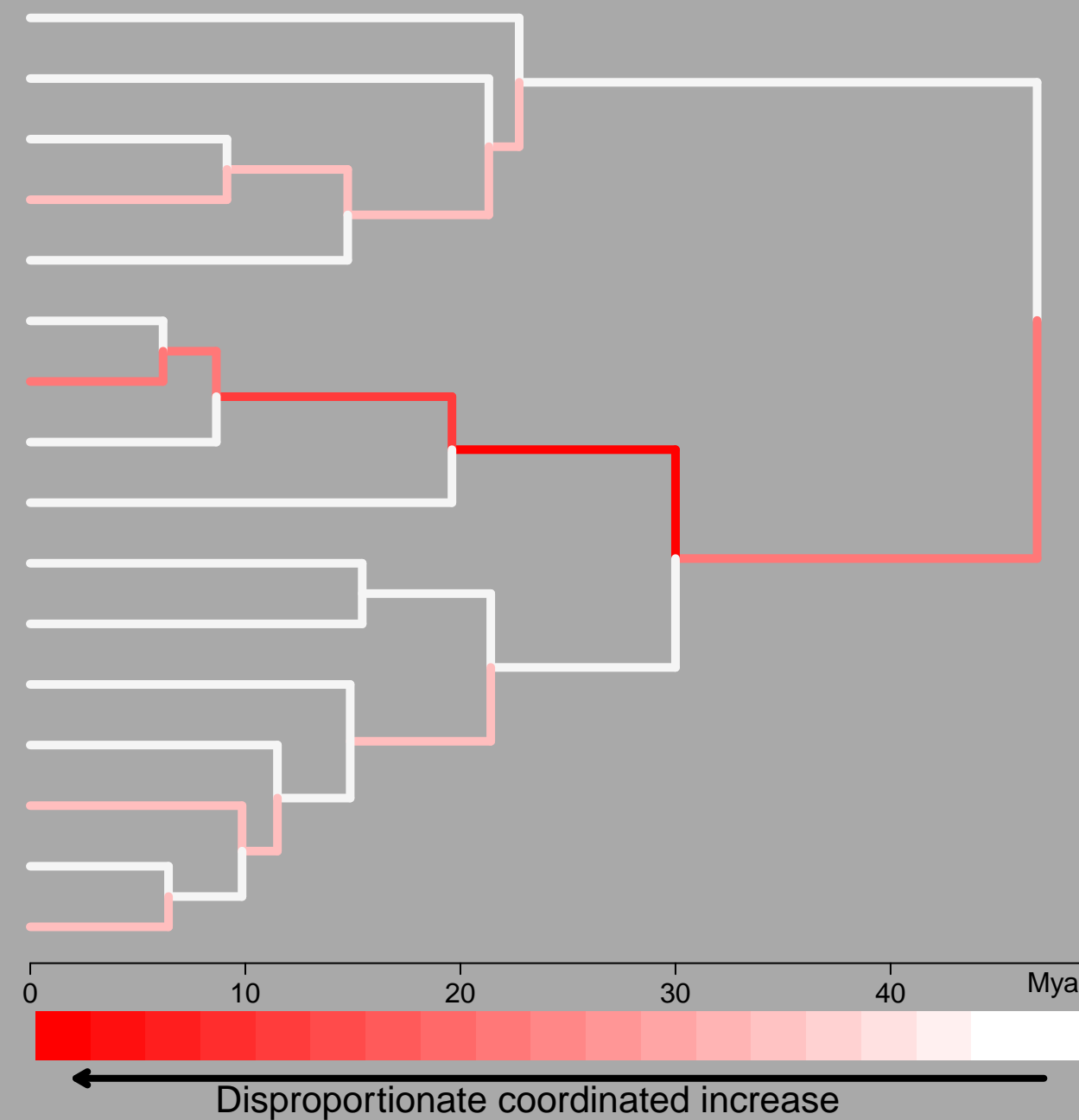

**(c) PF relative to FM and PCH**

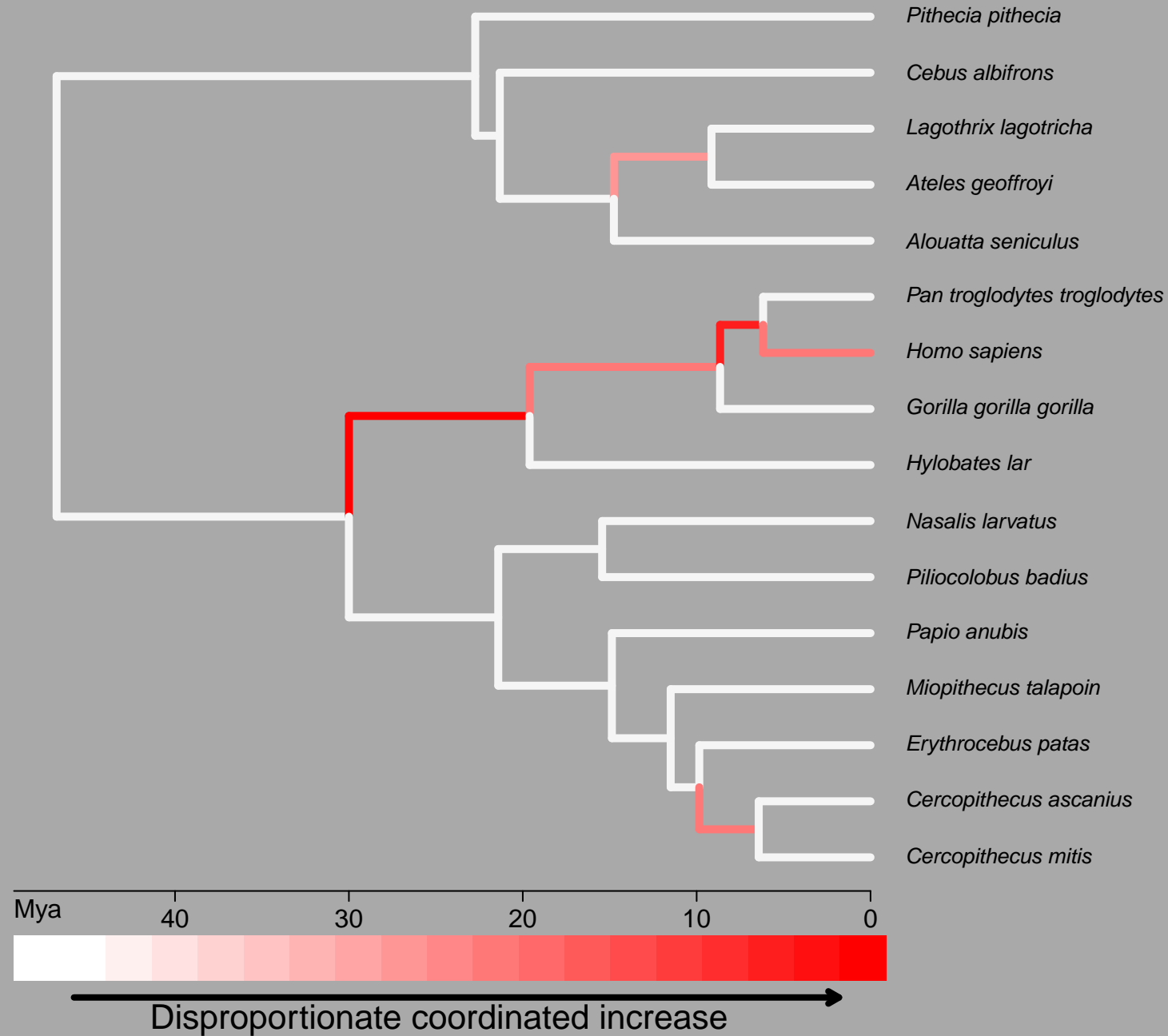

Supplement: Supplementary file 3 — Figure S3. As in Figure 2, results comprise the analyses of left cortical (PF and FM) and right cerebellar (PCH) hemispheres, and of right cortical (PF and FM) and left cerebellar (PCH) hemispheres. [file nyas1288-0059-sd3.pdf]

**(a) FM and PCH**

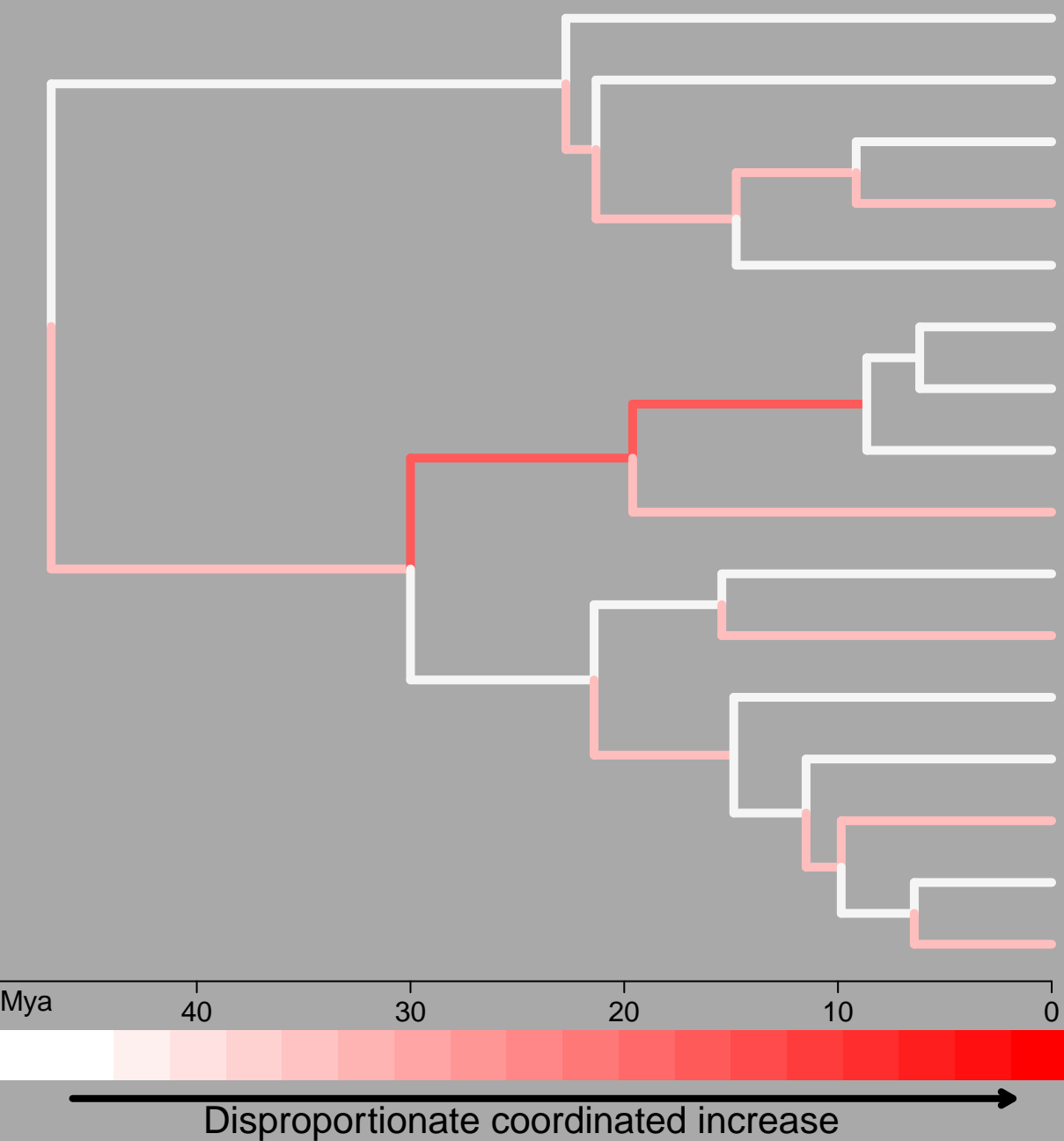

**(b) PF and PCH**

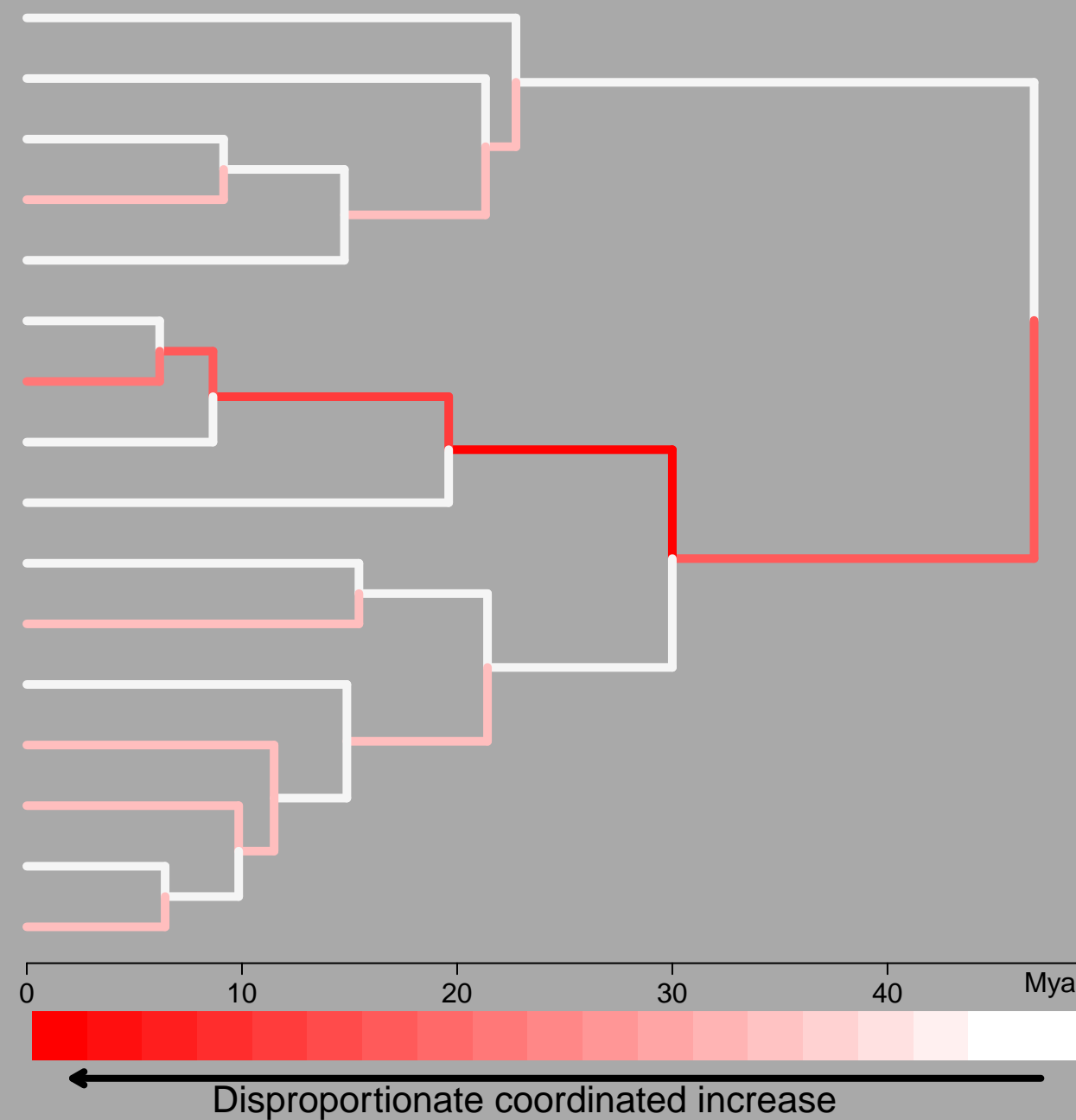

**(c) PF relative to FM and PCH**

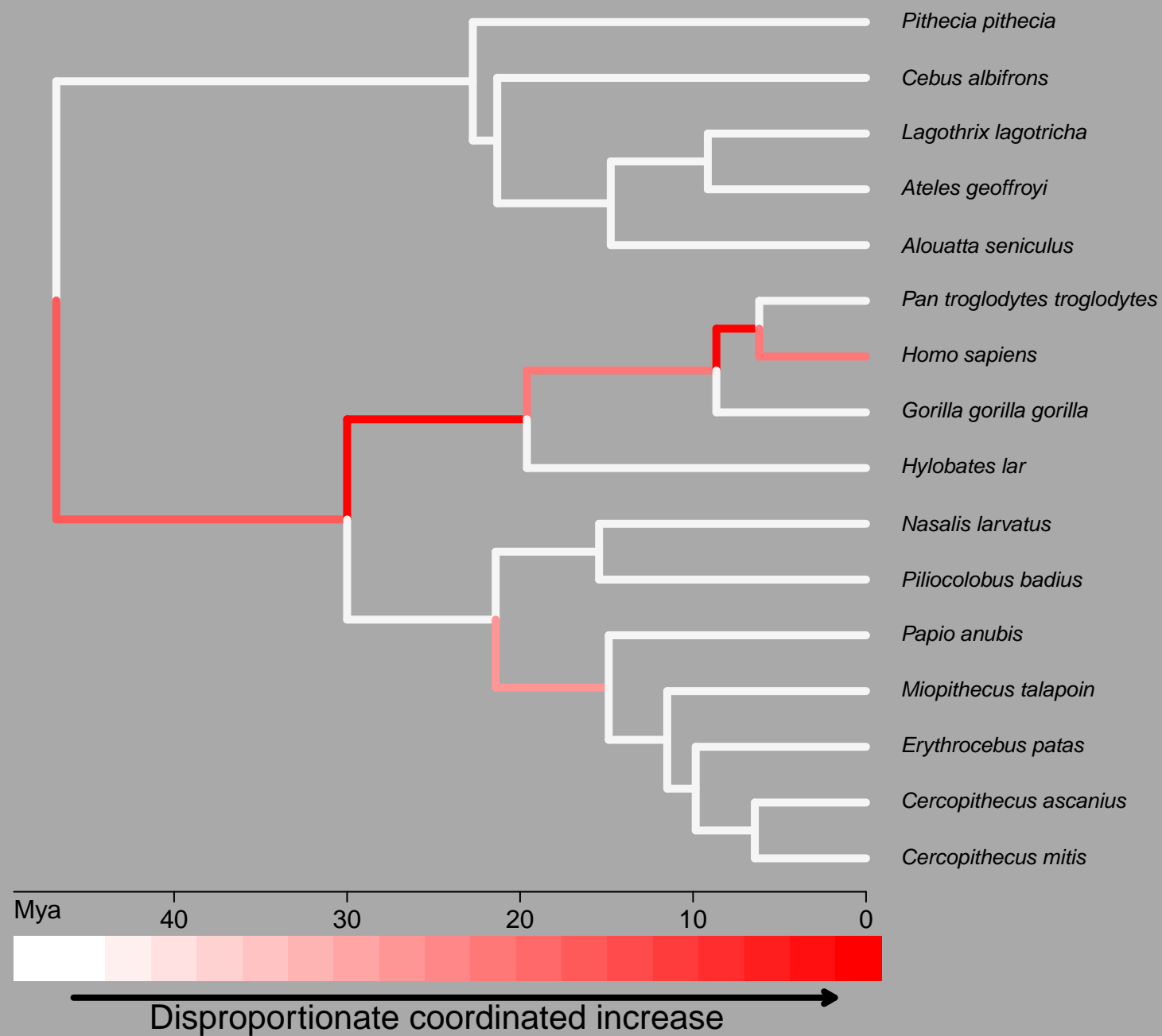

Supplement: Supplementary file 4 — Figure S4 [file nyas1288-0059-sd4.pdf]
